# Adult-onset Langerhans cell histiocytosis with multisystem involvement: A rare case report

**DOI:** 10.1016/j.radcr.2024.11.069

**Published:** 2024-12-11

**Authors:** Yaman M․ Alahmad, Omar Al Mukdad, Ahmad Huneity, Sarah Sayed, Renan Adam, Alaa Al-Taie

**Affiliations:** a Radiology Department, Hamad Medical Corporation, Doha, Qatar; b Pathology Department, Hamad Medical Corporation, Doha, Qatar

**Keywords:** Langerhans cell histiocytosis, Back pain, Neurologic deficits, Multisystem involvement, Diagnostic imaging, Histopathology

## Abstract

Langerhans cell histiocytosis (LCH) is a rare disorder, especially among adults, characterized by abnormal accumulation of dendritic histiocytes in various tissues, presenting as either single- or multi-system disease. In adults, spinal involvement is less common than long bone, while central nervous system manifestations, such as pituitary gland enlargement and stalk thickening, affect about a quarter of adult patients and may lead to significant endocrine disorders. Salivary gland involvement is another extremely rare manifestation of LCH. We report a 46-year-old lady who presented with back pain along with sensory and motor deficits in the lower limbs. Her blood pressure was elevated. Computed tomography angiography spotted a T6 vertebral body compression fracture. Magnetic resonance of the spine showed T6 vertebral body pathological collapse with epidural soft tissue component compressing the spinal cord. Other imaging modalities showed features suggestive of central nervous system and salivary gland involvement. She underwent T4-T8 pedicle screws fixation with T6 decompressive laminectomy and biopsy of the epidural lesion, which revealed histopathological features of LCH. This is an extremely rare case of adult-onset LCH with multi-system involvement: musculoskeletal, salivary and central nervous systems. This case could serve as a crucial reference for both clinicians, radiologists and researchers, enriching the existing knowledge base on adult-onset LCH.

## Background

Langerhans cell histiocytosis (LCH) is a rare proliferative disorder characterized by the clonal accumulation and infiltration of dendritic histiocytes within various tissues causing a single- or multi- system disease [1]. The exact cause of LCH remains unknown [2]. The incidence of LCH in adults is notably lower than in children, with estimates ranging from 1 to 2 cases per million compared to 5–10 cases per million children per year [3]. While bone involvement is the most frequent, about 50% of cases, spinal manifestations are less common [4]. LCH spine involvement often clinically manifests as neck or back pain, restricted motion of spine and/or neurologic deficits [5]. LCH central nervous system involvement, particularly pituitary gland enlargement and stalk thickening, occurs in approximately 25% of adult cases and may lead to diabetes insipidus or panhypopituitarism [6,7]. Salivary gland involvement is another rare manifestation of LCH with only a few reported cases of parotid glands involvement [8].

## Case presentation

**Fig. 1:**
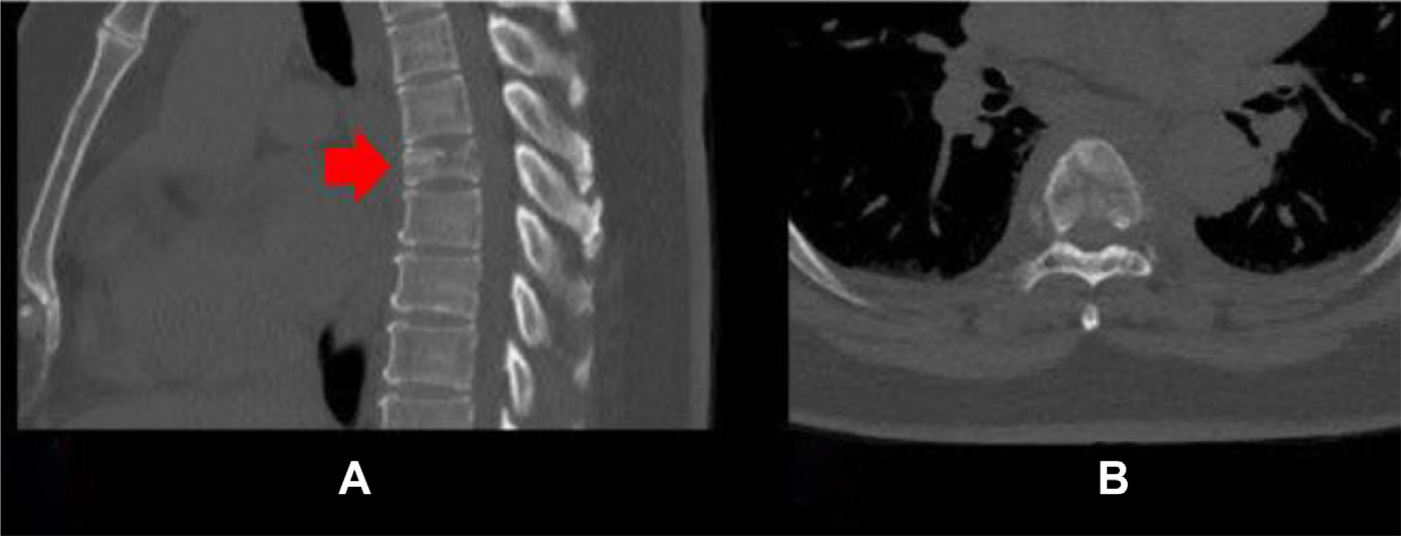
Selected thoracic region sagittal (A) and axial (B) images of computed tomography scan -bone window- revealed T6 vertebral body partial collapse with suspicion of an underlying bone lesion (red arrow).

A 46-year-old female, with no significant medical or surgical past medical history, presented to the emergency department (ED) with 2-week history of severe progressive back pain radiating lower limbs along with bilateral lower limbs weakness and numbness. Physical examination revealed thoracolumbar tenderness, bilateral paraplegia, and bilateral positive Babinski signs. Her blood pressure was elevated 200/110 mmHg with normal body temperature and results of initial blood tests revealed slightly raised inflammatory markers, white blood test (WBC) 10.6 × 10^3/uL [normal range: 4.0-10 x 10^3/uL] and C-reactive protein (CRP) 27.6 mg/L [normal range: 0-5 mg/L]. A computed tomography (CT) head scan ruled out acute hemorrhagic stroke and a CT angiography of the aorta excluded aortic dissection but spotted a T6 vertebral body compression fracture (Fig. 1).

**Fig. 2:**
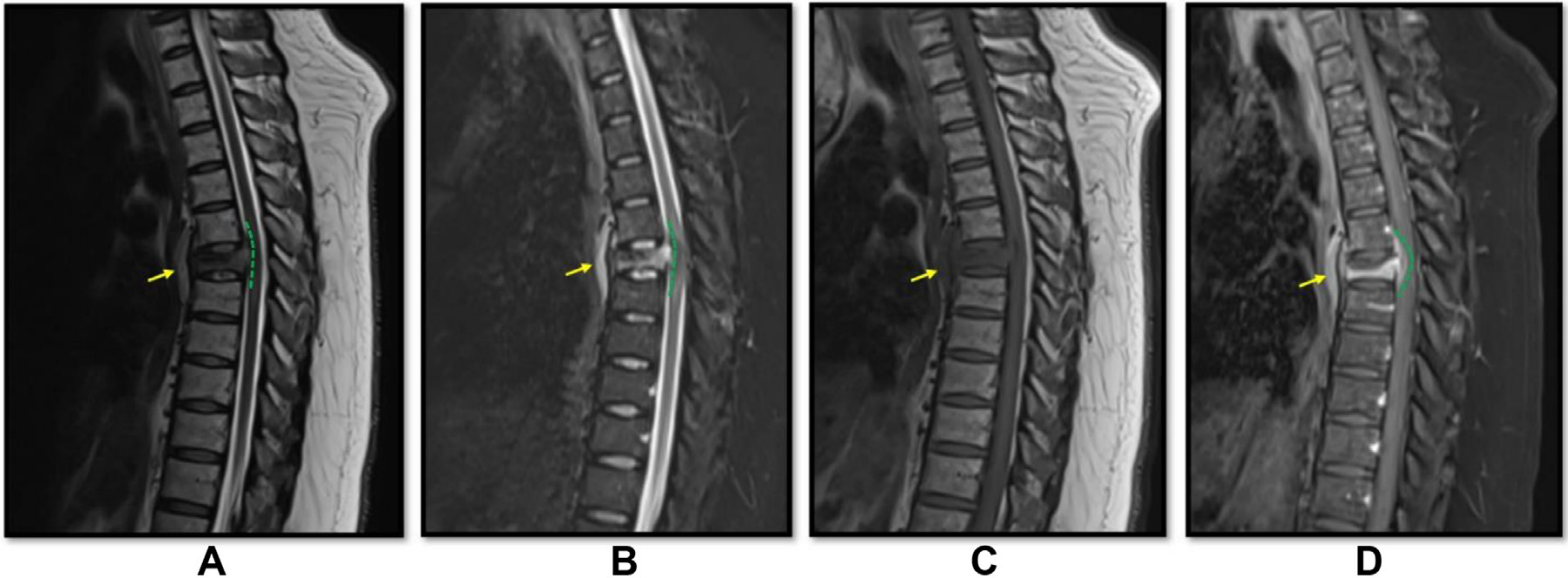
Selected images of multiplanar multisequence MRI of thoracolumbar spine with intravenous contrast. Sagittal planes: (A) T2 (B) STIR (C) T1 (D) T1 (D) T1 post contrast. There is T6 vertebral body collapse with underlying abnormal marrow signal of T1 low and T2/STIR bright signal and heterogenous postcontrast enhancement. Element of posterior retropulsion as well as associated prevertebral, paravertebral and retrovertebral intraspinal ventral epidural enhancing soft tissue component at the same T5-T6 level (green dotted line) with moderate cord compression.

Magnetic resonance imaging (MRI) of the thoracolumbar spine revealed intraspinal ventral epidural enhancing soft tissue lesion within the pathologic fracture of T6 vertebral body, along with moderate cord compression at the same level (Fig. 2).

**Fig. 3:**
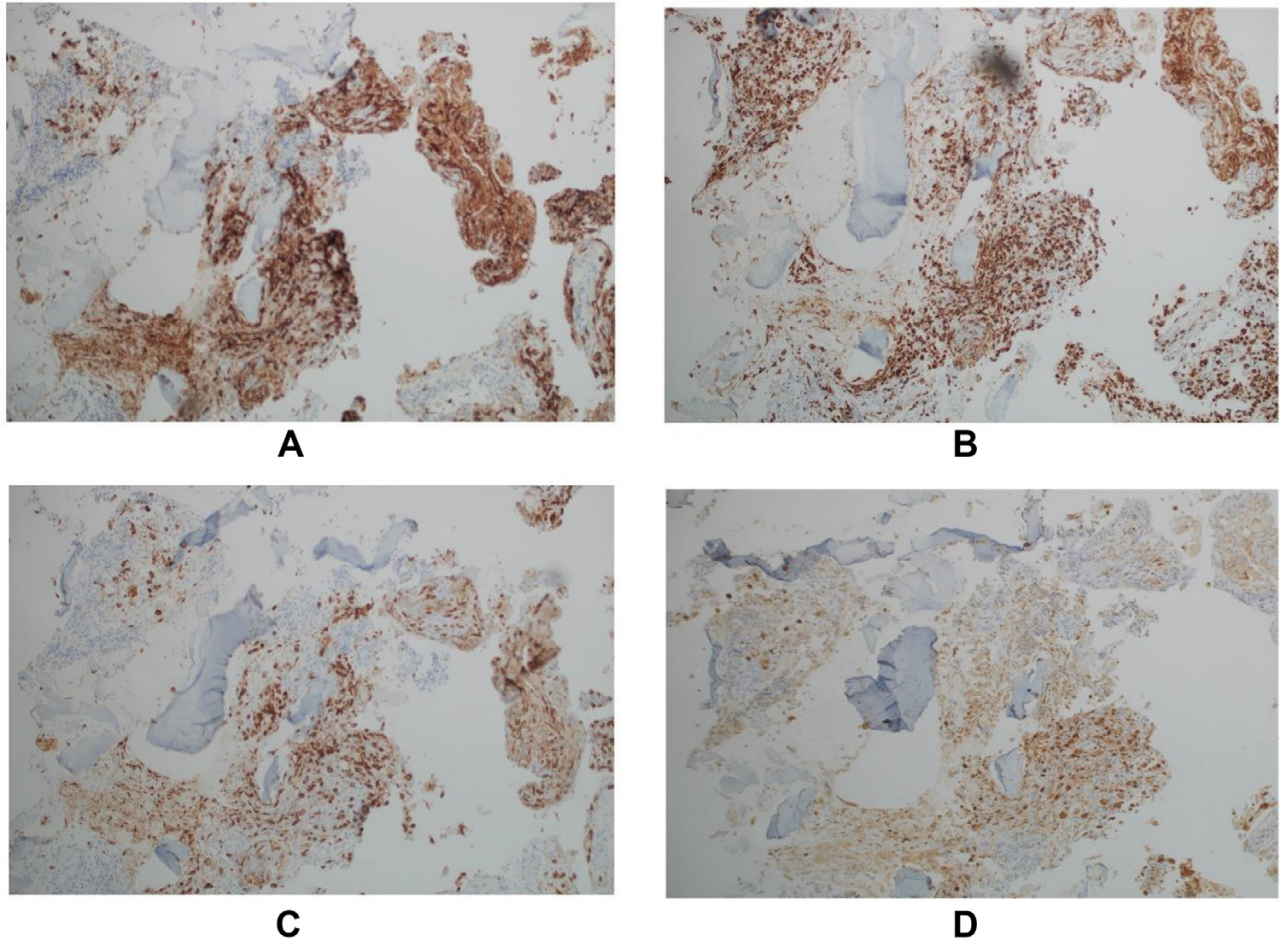
Histopathology examination. (A) CD1a, (B) CD68, (C) Langerin, (D) S100.

**Fig. 4:**
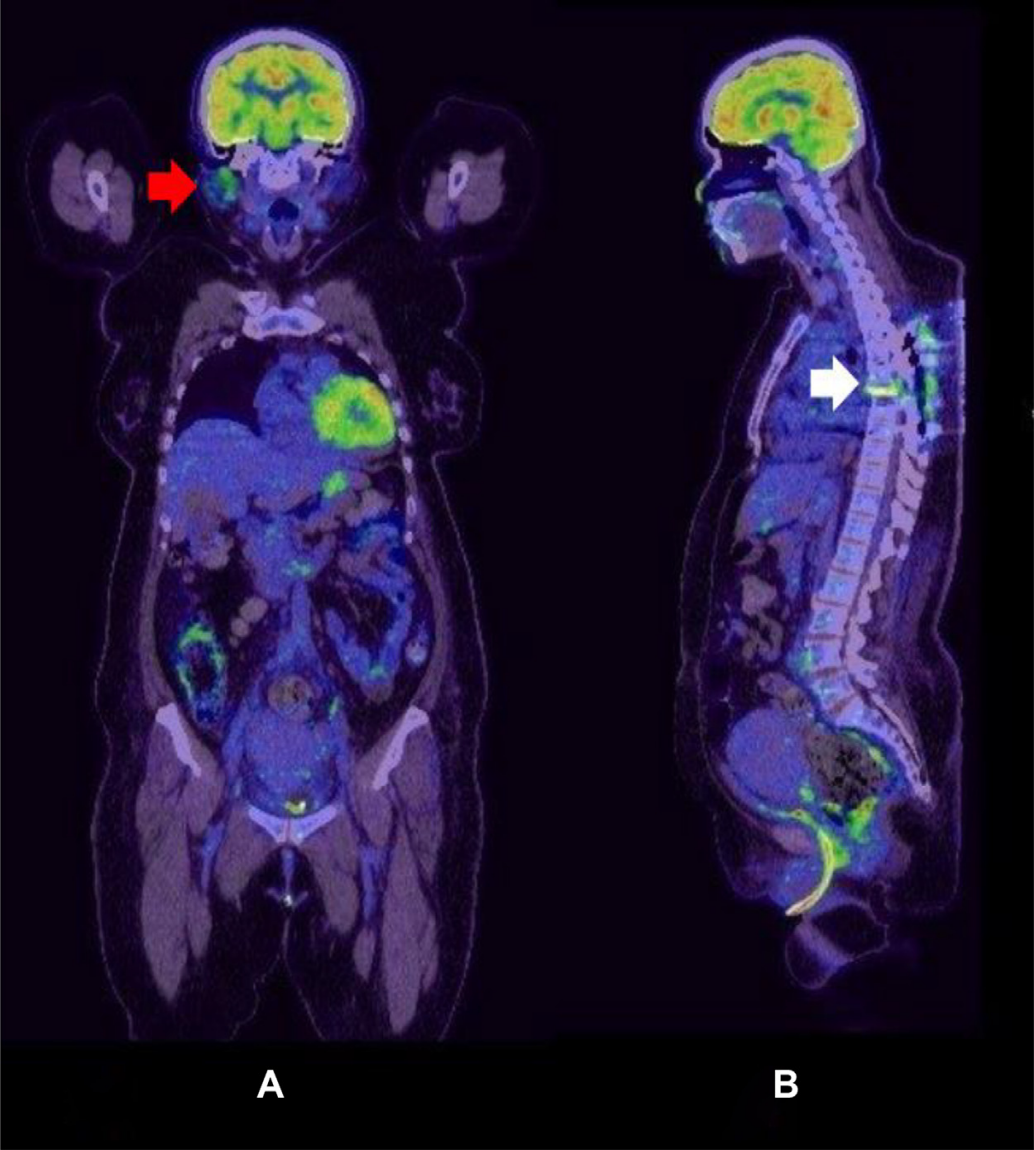
Whole body positron emission tomography. (A) Coronal plane showing asymmetric right-sided parotid gland high uptake (red arrow). (B) Sagittal plane showing mild increased uptake at the area of T6 vertebra consitent with post operative changes. Note the T4-T8 interpedicular screw fixation artefact at mid-thoracic spine.

**Fig. 5:**
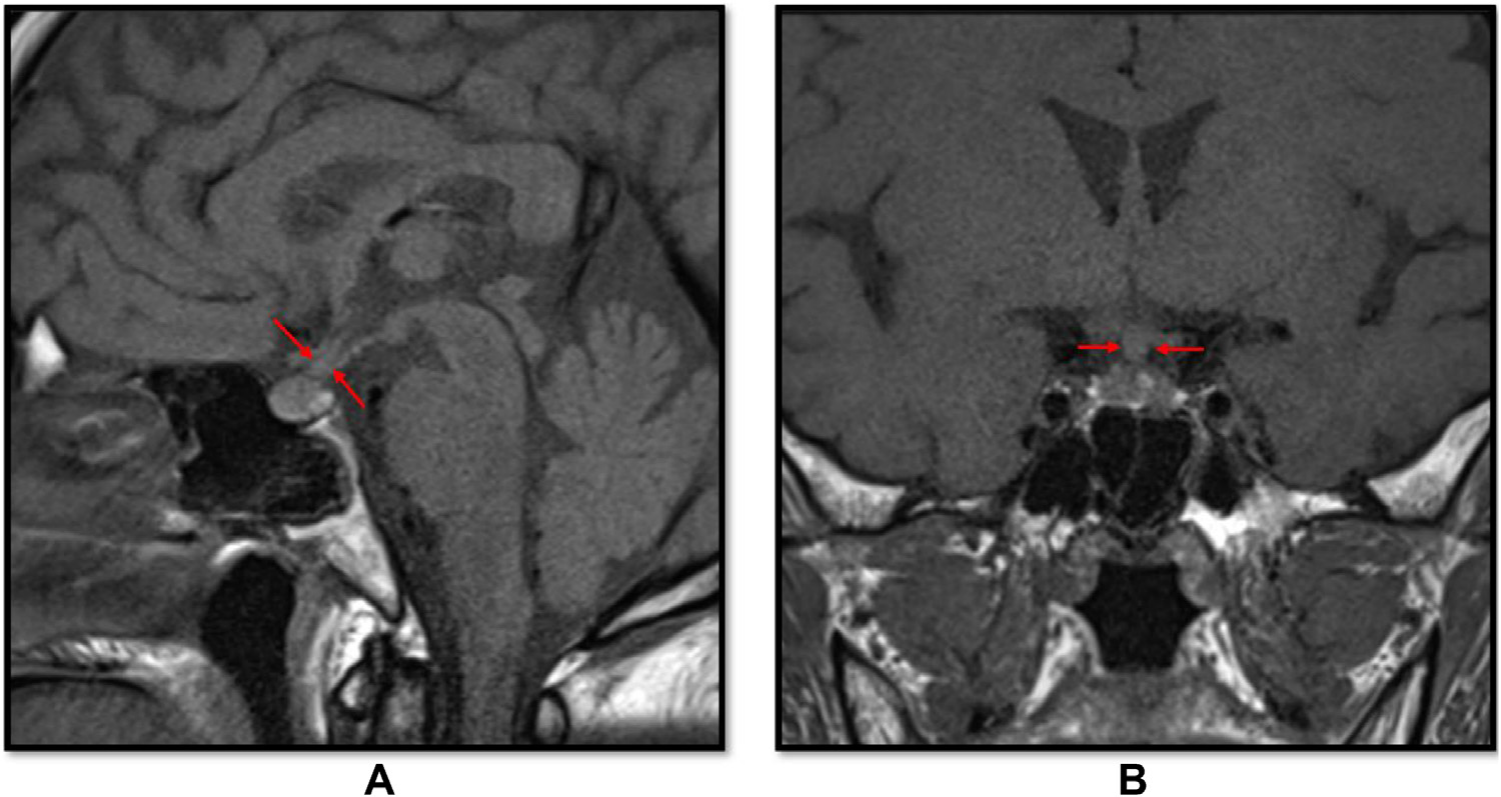
Selected T1 sagittal (A) and coronal (B) images of multiplanar multisequence MRI of pituitary gland without intravenous contrast (avoided due to acute kidney injury), showing mild thickening of the pituitary stalk (red arrows). The width of pituitary stalk measured 4 mm.

Due to the patient neurological deficits and radiologic finding, the patient underwent T4-T8 pedicle screw fixation, T6 decompressive laminectomy, and biopsy of the epidural lesion. Histopathological analysis revealed findings consistent with LCH, with positive immunohistochemical staining for CD1a, langerin, S100, and CD68 (Fig. 3). BRAF mutation was negative. Screening whole body positron emission tomography (PET) CT scan for metabolic assessment showed increased uptake at the area of T6 vertebra, suggestive of residual/active disease, and high right parotid gland uptake (Fig. 4). MRI of the head showed a thickened pituitary stalk (Fig. 5). Accordingly, endocrine assessment was made, which was unremarkable.

## Discussion

LCH is more prevalent in the pediatric population, with the highest incidence age range between 1-15 years [9,10]. Males are more probable to manifest the disease with a ratio of (M: F, 2:1) [11]. Clinical and radiological findings of LCH are nonspecific [12]. The diagnosis of LCH is solely based on histopathological examination. The histopathological pattern demonstrates a diffuse infiltration of pale staining mononuclear cells that resemble histiocytes with indistinct cytoplasmic borders and rounded or indented vesicular nuclei [13]. Given its rarity among adults, it is unlikely to be listed on initial encounter management differential diagnoses.

In regard to LCH of the spine, radiologically a single or multiple well-defined osteolytic lesions accompanied by soft tissue lesions leading to vertebral collapse/flattening -vertebra plana- are the most common radiologic finding [12].

The neurological manifestations of LCH are variable [14]. Diabetes insipidus (DI) is the most common manifestation of CNS involvement, which can cause frequent urination [14]. Co-existing anterior pituitary endocrinopathies is common among LCH related DI patients [14]. Radiologically, pituitary gland enlargement and stalk thickening are nonspecific but well-established [7]. Pituitary stalk width of 4 mm or greater is commonly used as a cutoff to point towards pathological thickening [15].

Even though the reported patient did not complain of parotid gland and no palpable mass was observed at the expected anatomical location, fluorodeoxyglucose (FDG) high uptake of parotid gland is likely to be explained by early systemic involvement of LCH. There have been several case reports of LCH with parotid glands involvement of variable presentation; isolated, multisystem, unilateral and bilateral [8,[16], [17], [18]]. Isolated LCH of sublingual gland was also reported [8].

In short, this is a very rare case of an adult lady who was presented with back pain along with sensory and motor deficits in the lower limbs. MRI spine showed T6 vertebral body pathological collapse with epidural soft tissue component compressing the spinal cord. She underwent T4-T8 pedicle screws fixation with T6 decompressive laminectomy and biopsy of the epidural lesion, which revealed histopathological features of LCH. Other imaging modalities showed findings suggestive of multi-system involvement.

## Patient consent

Written informed consent for publication was obtained from the patient.
